# Endoscopic versus open surgery for soft tissue vascular anomalies and benign tumors: a two-center propensity score-matched study

**DOI:** 10.3389/fsurg.2026.1733095

**Published:** 2026-04-15

**Authors:** Ming Li, Huaijie Wang, Zhengtuan Guo, Chong Xie, Weilong Lin, Peihua Wang, Weijia Yang, Lingling He, Lijuan Zhang

**Affiliations:** 1 Department of Burn & Plastic Surgery, The Second Affiliated Hospital to Shandong First Medical University, Tai’an, China; 2 Department of Pediatric Surgery & Vascular Anomalies, Xi’an International Medical Center Hospital, Xi’an, China

**Keywords:** endoscopic surgery, FAVA, lipoma, *PTEN*, surgery, vascular malformation

## Abstract

**Background:**

No large-scale study has compared the outcomes of soft-tissue endoscopic surgery (SOFTES) with conventional open surgery (OS) for vascular anomalies and benign soft tissue tumors. We aimed to compare the operative safety, efficacy, and outcomes of the two approaches for soft tissue lesions.

**Methods:**

Patients (*n* = 414) undergoing SOFTES or OS in two centers were retrospectively reviewed. Propensity score matching was used to minimize selection bias and group differences. The treatment outcomes were compared between the two groups.

**Results:**

After matching, 150 patients (SOFTES: 75; OS: 75) were included. Compared with the OS group, the estimated blood loss in the SOFTES group was significantly lower [20 mL (1-500) vs. 50 mL (5-600); *p* = 0.001]; however, the operative duration was significantly longer [223 minutes (35-490) vs. 173 minutes (37-494); *p* = 0.008]. Major complications were not observed. The incidence of focal burn of skin in the SOFTES group was higher than that in the OS group (*p* = 0.048). The incidences of superficial peroneal nerve injury, postoperative bleeding, persistent lymph drainage, local sensory paralyses (<5 cm^2^), surgical site infection, hematoma/seroma, and residual mild pain were not significantly different between the two groups. However, the incidence of wound dehiscence [*n* = 0 (0.00%) vs. *n* = 7 (9.33%); *p* = 0.010] and flap necrosis [*n* = 1 (1.33%) vs. *n* = 11 (14.67%); *p* = 0.005] was significantly higher in the OS group than in the SOFTES group. Residual pain was cured or significantly improved. All patients achieved resolution of contracture had normal or near-normal joint motion.

**Conclusions:**

Endoscopic surgery is a safe and effective treatment option for various vascular anomalies and benign soft tissue tumors in selected patients. This paradigm shift has many advantages in terms of clinical outcomes and reduces postoperative complications.

## Introduction

Soft tissue lesions refer to lesions located in the soft tissues of the somatic compartments, and include subcutaneous, fascial, intermuscular, and intramuscular lesions (1). Some can be managed with nonsurgical treatments, such as embolization (2), sclerotherapy (3), and medical therapy for some vascular tumors (4–7) and malformations (8). Nevertheless, surgery is the most common elective treatment for soft tissue lesions such as lipoma (9, 10), fibroma (11), dermoids (10), fibro-adipose vascular anomaly (FAVA) (8, 12, 13), microcystic lymphatic malformation (mLM) (1, 3, 12, 14–16), venous malformation (VM) with thrombosis and contracture (1, 12), primary lymphedema (1), and other benign tumors (1, 4), Historically, open surgery has been a lengthy treatment and long linear surgical scars usually lead to significant aesthetic concerns and scarring contracture (17–19). With the development of minimally invasive techniques for the treatment of soft tissue diseases, endoscopic surgery has been used (1, 8–10, 13, 19, 20), from multi-portal surgery to single-site endoscopic surgery (1, 8, 9, 12, 13). The soft tissue spaces of the body, including the subcutaneous and intermuscular spaces, provide a broad operating field for endoscopic surgeons (13, 21). We have performed endoscopic surgery to resect various benign soft-tissue lesions using laparoscopic devices since 2016 (12, 15). We previously reported endoscopic surgery as a minimally invasive, effective, and safe treatment for subcutaneous and intramuscular lesions, and extensive vascular malformations, including mLM (12), VM (12), superficial venous thrombosis (SVT) (12), adipose overgrowth with vascular malformation (12), marginal vein in Klippel-Trénaunay syndrome (KTS) (12), FAVA (8, 12, 13), primary lymphedema and dermatofibrosarcoma protuberans (1), Kaposiform hemangioendothelioma (1), capillary malformation-arteriovenous malformation (CM-AVM) (22), and verrucous venous malformation (VVM) (22). We called these resections soft tissue endoscopic surgery (SOFTES). Although some recent studies (13, 19) have verified the efficacy, feasibility, safety, reduced incisional complications, shortened recovery time, and cosmetic results of SOFTES, these results were based on reports from small case cohorts. Strict comparisons between SOFTES and open surgery (OS) in larger cohorts are still lacking. Thus, in this comparative study, which included various soft tissue lesions, we aimed to evaluate the advantages and disadvantages of SOFTES and OS.

## Methods

### Patients

We reviewed patients who underwent OS or SOFTES for soft tissue lesions at any site. Given the retrospective nature of this study and the clinical preference for endoscopic approaches in selected cases at our center, a prospective randomized controlled trial was not feasible. Therefore, we conducted a retrospective two-center cohort study to compare SOFTES and OS using propensity score matching to mitigate selection bias. This study enrolled patients who underwent OS between April 2023 and December 2024 at another center, and between September 2019 and July 2021 at our center. Patients undergoing SOFTES were recruited between August 2021 and April 2024. Patients undergoing OS were enrolled from earlier time periods (April 2023–December 2024 at one center; September 2019–July 2021 at the other) to reflect the era before SOFTES became routine, whereas SOFTES patients were enrolled from August 2021 onward. This temporal separation was intended to minimize the influence of evolving surgical preferences and to allow for meaningful comparison between established techniques.

**Figure 1:**
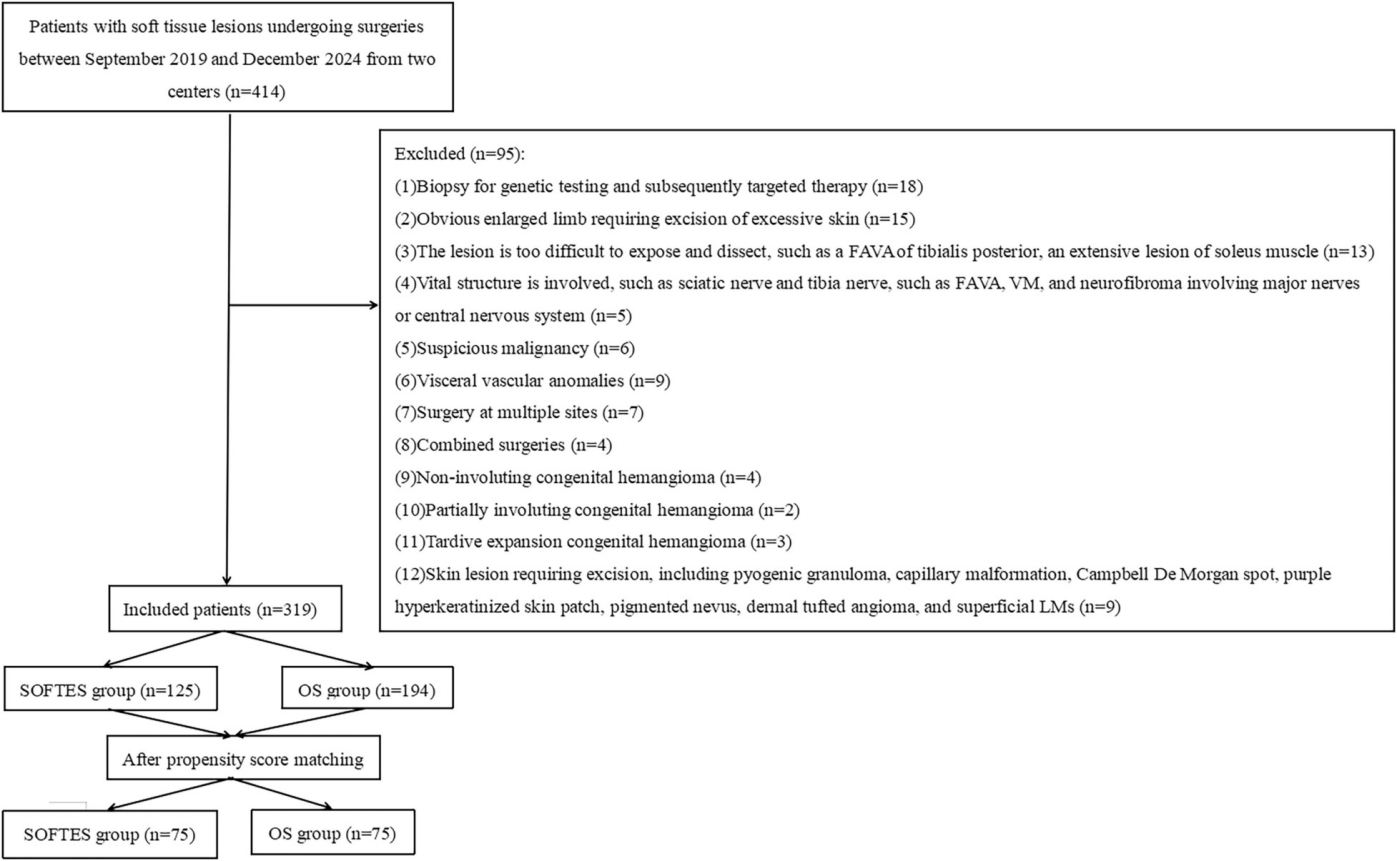
Flowchart of patient selection.

Exclusion criteria were applied to ensure that both surgical approaches were clinically appropriate and technically comparable. These included: (1) cases requiring plastic skin removal or grafting (12), as these often involve complex reconstruction not relevant to the comparison; (2) biopsy-only procedures; (3) lesions involving the central nervous system; (4) extensive deep muscular infiltration where neither approach would be curative, or lesions infiltrating vital structures (e.g., the sciatic and tibial nerves); and (5) lesions deemed too difficult to expose endoscopically or openly (e.g., posterior tibialis lesions and an extensive lesion of the soleus muscle). These exclusions were intended to create a more homogeneous cohort for comparison. The patients selection flowchart is shown in Figure 1. Owing to the non-randomized nature of this study, 1:1 propensity score nearest-neighbor matching was applied to minimize bias in patient selection between the two groups. The propensity scores for each patient were generated using a multivariate model, with matching factors including age, presence of comorbidities, diagnoses, surgical sites, lesion size and depth, operative time, blood loss, intra- and postoperative complications at the initial assessment.

Written informed consent was obtained from all parents or guardians before the procedures. The potential advantages and disadvantages of SOFTES versus the OS are discussed. The ethics committees of the two centers approved the study protocol (approval numbers: 2025097 and 2025-H-053). The study was conducted in accordance with the ethical principles of the Declaration of Helsinki. Data were extracted from the hospital medical record databases, including sex, age, diagnosis, site of surgery, surgery, operative duration, estimated blood loss, and follow-up.

### Indications of SOFTES

Prior to SOFTES, ultrasonic examination and magnetic resonance imaging were performed in all patients to delineate the extent of lesion formation and identify the involved structures. Diagnosis and treatment decisions were made by a multidisciplinary team comprising pediatric surgeons, ultrasonologists, and pediatric interventional radiologists. If an unsatisfactory response was predicted or failure occurred after nonoperative therapy in patients with primary lymphedema, mLM, VM with fibrosis, and contracture, surgery was recommended. Non-operative therapy included anticoagulant medication, compressive elastic wrap, and interventional sclerotherapy with ethanol, polidocanol, and bleomycin. Surgery was also recommended for patients with contractures, functional impairments, and solid soft tissue lesions.

The indications and general principles of SOFTES are listed in Table 1.

**Table 1:** Indications and principles for soft tissue endoscopic surgery.

Indications	Principles for endoscopic surgery
Lipoma	Radical resection
Intramuscular capillary-type hemangioma	Radical resection
Primary lymphedema	Debulking
*PTEN* (type) hamartoma of soft tissue	Biopsy; Radical resection; Debulking
CM-AVM with adipose overgrowth	Debulking
Classic VVM with subcutaneous expansion	Resecting subcutaneous part
VVM - subcutaneous variant	Radical resection
SVT in KTS	Thrombectomy
VM with thrombosis and fibrosis and contracture	Radical resection; Debulking; Relaxation
Fibro-adipose vascular anomaly (stage 1/2)	Radical resection; Transverse gastrocnemius aponeurotic recession; Achilles tenotomy; Relaxation of ankle capsule; Neurolysis/neurectomy; Tendon transfer
Microcystic LM	Debulking; Radical resection if possible
Mixed cystic LM	Debulking
Fibroma	Radical resection
Diffuse capillary malformation with overgrowth	Adipose debulking
Sequela of tufted angioma or Kaposiform hemangioendothelioma	Resecting subcutaneous part; Debulking
Other benign soft tissue lesions	Radical resection

CM-AVM, capillary malformation-arteriovenous malformation; VVM, verrucous venous malformation; SVT, superficial venous thrombosis; KTS, Klippel-Trénaunay syndrome; VM, venous malformation; LM, lymphatic malformation.

### Relative contraindications of SOFTES

**Figure 2:**
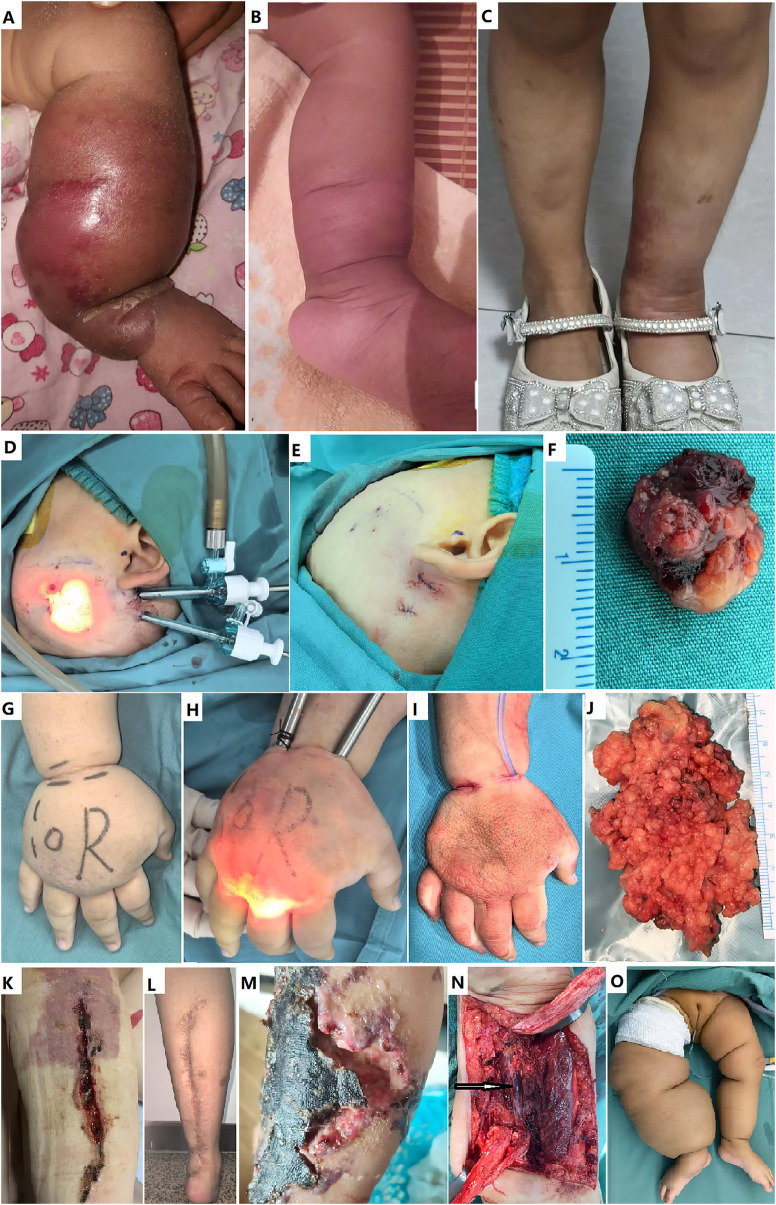
Techniques of soft tissue endoscopic surgery and some complications of open surgery. **(A-C)** A female of one month old was diagnosed as kaposiform hemangioendothelioma with the Kasabach-Merritt phenomenon, and successfully managed with oral sirolimus for one year. However, lymphedema persisted as the sequela. At the age of three, she underwent open debulking of the lymphedema and recovered uneventfully. **(D-F)** Endoscopic resection of an intra-masseter venous malformation with thrombosis. **(G-J)** Endoscopic debulking of primary lymphedema at the opisthenar. **(K)** Wound dehiscence after open debulking adipose-overgrowth. **(L)** A long linear scar with severe contracture after open resection of an extensive venous malformation in the calf at another hospital. **(M)** Flap necrosis after an open surgery. **(N and O)** Sciatic nerve involvement (the arrow), and microcystic lymphatic malformation with excessive skin are contraindicated for endoscopic resection. The excessive skin required open plastic excision.

**Figure 3:**
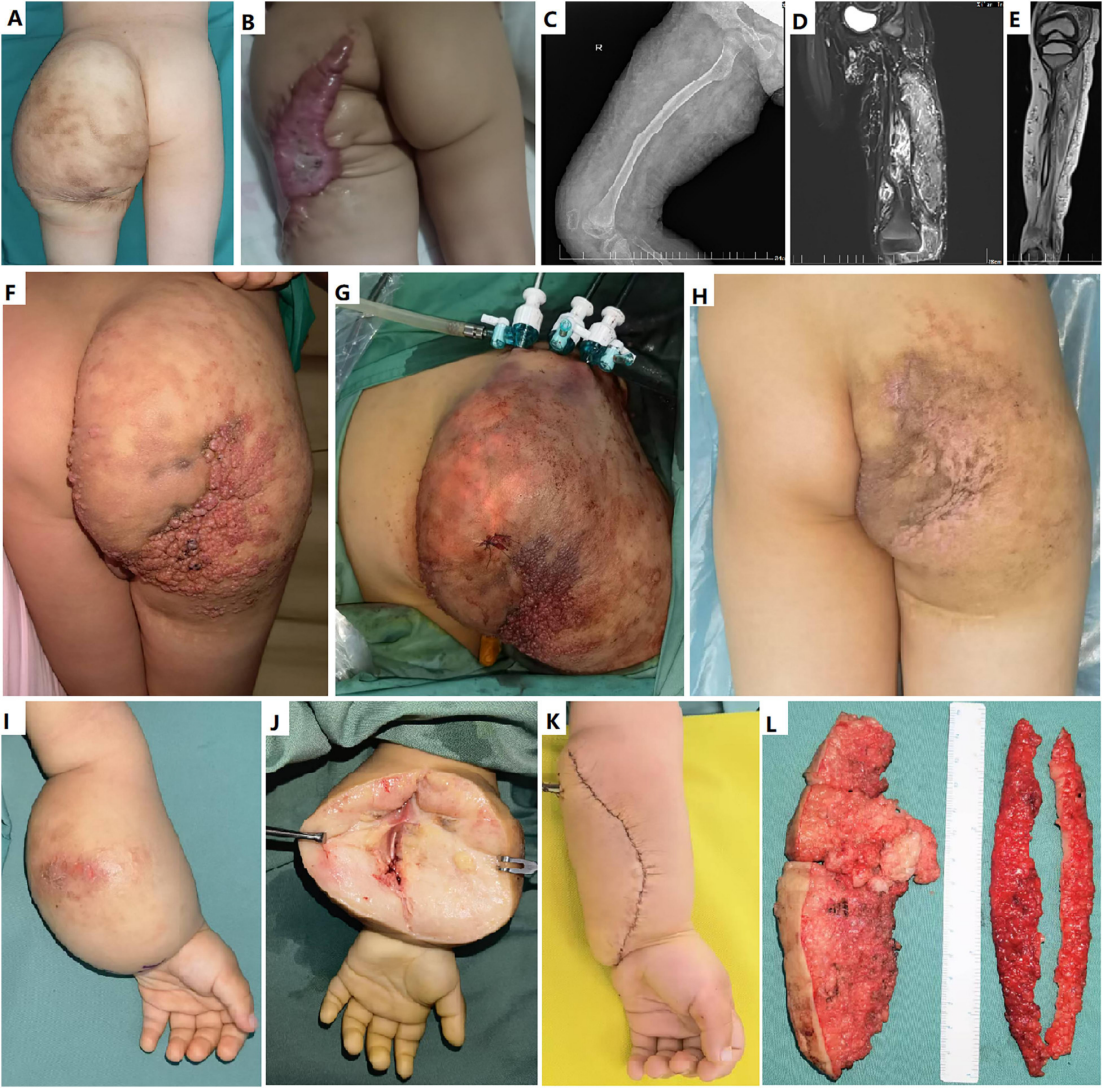
Relative contraindications for soft tissue endoscopic surgery and some postoperative complications. **(A and B)** A boy with microcystic lymphatic malformation with excessive skin of gluteal region underwent open debulking and skin excision. Postoperative scarring was obvious. **(C-E)** An extensive *PTEN* (type) hamartoma of soft tissue involved all or most muscles of the calf and thigh in a boy, both endoscopic and open resection was absolutely contraindicated. **(F-H)** Endoscopic debulking of microcystic lymphatic malformation with mild excessive skin of gluteal region in a boy. Additional curettage and sclerotherapy technique was used to manage the superficial lesions. **(I-L)** A primary lymphedema with excessive skin of the forearm was contraindicated for endoscopic debulking. She underwent open debulking and skin excision.

If an extensive or diffused lesion, such as FAVA or VM, involved all or most muscles of the calf, thigh, or arm, excision surgery, both SOFTES and OS was absolutely contraindicated (Figures 2, 3). Relative contraindications of SOFTES (Figures 2, 3) include: (1) obvious enlarged limb requiring excision of excessive skin; (2) common VM, which is predicted to respond well to sclerotherapy; (3) macrocystic or mixed cystic LM, which is predicted to respond well to sclerotherapy; (4) lesions that are too difficult to expose and dissect, such as a lesion of the tibialis posterior, an extensive lesion of the soleus muscle, or involvement of vital structures, such as the sciatic and tibial nerves; and (5) suspicion of malignancy. Decisions were made by our multidisciplinary team.

### SOFTES technique

Preoperative ultrasound examination was used to detect and delineate the extent of the lesions and provide body surface markers of vital nerves and vessels. Magnetic resonance imaging was used to confirm the location of the lesion and the involved structures. The surgery was performed under general anesthesia. The patient was positioned to optimize the approach to and exposure of the lesion. Before surgery was initiated in the lower extremities, a tourniquet (25–45 kPa) was employed to prevent bleeding and gas or fat embolism, with a duration of 60–90 min and an interval of 15–20 min. If the lesion was located in the gastrocnemius and its fascia, or in the soleus, the prone position with the distal limb elevated was preferred. Small incisions were made just above the distal Achilles tendon, medial malleolus, and/or lateral malleolus. A vascular tunneler was introduced and used to bluntly develop the initial cavernous workspace and enlarge the subcutaneous space. Two or three 5-mm trocars were introduced into the initial workspace. Most calf lesions can be successfully resected using this access (13).

If the lesion was located on the anterior leg, the supine position was optimal to expose the lesion. Portal incisions at the distal Achilles tendon, medial malleolus, and/or lateral malleolus are classic access points. These incisions can be concealed by socks. If the lesion was in the fascia or muscles of the thigh, whether a tourniquet was used depended on the lesion. A port incision was made 5-10 cm beyond the lesion site. In cases with lesions on the trunk, face, or neck, tourniquet use was impossible. Preemptive transcutaneous ligation of the main vessels, according to preoperative ultrasonic body surface projections, are an alternative to limit bleeding (22). For lesions of the face, such as intra-masseter venous thrombi, 3-mm trocars were preferred (Figure 2). Incisions were made behind the ear or at submandibular sites.

Carbon dioxide was insufflated into the initial workspace through a port at a low flow rate to maintain a pressure of 6–12 mm Hg. A 30° endoscope was then placed through a port to visualize the separation of the subcutaneous nonvital neurovascular bundles and to expose the lesion using a monopolar hook-endocautery or an ultrasonic scalpel. The skin was then elevated to enlarge the workspace for dissection. Silk sutures were used for lesions or muscle traction, if required. Intraoperative repeated rinsing with saline improved the visual field.

The thickened deep fascia was resected in patients with subcutaneous vascular malformations. When debulking lymphedema or extensive vascular malformation, the full thickness of the skin flap was elevated, but as thinly as possible, to one-third to half the circumference of the limb. If the lesion involved more than half the circumference of the extremity, staged surgery was performed to secure flap survival. The goal of surgery was to improve the mass effect and local contours. Radical resection was usually impossible, except for localized or small lesions, such as lipoma, fibroma, VVM-subcutaneous variant, stage 1/2 FAVA, intramuscular capillary-type hemangioma, and some SVTs. A tourniquet and preemptive percutaneous ligation of the main feeding arteries and drainage veins were used to prevent bleeding from arteriovenous malformation (22).

In patients with intramuscular lesions, such as FAVA and *PTEN* (type) hamartoma of soft tissue, the thickened deep fascia and affected muscles must be radically resected if possible, protecting and preserving vital nerves and blood vessels. Following lesion resection, endoscopic transverse gastrocnemius aponeurotic recession was performed if Achilles tendon lengthening was required. Achilles tenotomy, ankle capsule relaxation, neurolysis/neurectomy, and tendon transfers were also be performed with endoscopic assistance. The detailed surgical principle is based on individual disease staging (8, 13).

After specimen retrieval and surgical field rinsing, a suction drain was placed through the port incision if there was excessive dead space. After surgery, a compressive elastic wrap was routinely prescribed to prevent the collection of lymphatic fluid or blood beneath the flap and circumferential compression adhered the flap to the underlying tissue. The suction drain was selected as the access point for postoperative sclerotherapy if lymph drainage persisted after LM debulking. Additional technical details can be found in another reports (10, 12, 13, 19, 20).

### OS technique

The preoperative preparation and anesthesia administration for OS were similar to those for SOFTES. A tourniquet (25-45 kPa) was used to prevent bleeding. A linear incision of appropriate length was made just over the lesion. Hemostasis and irrigation of the surgical field were performed when bleeding occurred, and the lesion was excised as extensively as possible. However, according to Miller's principle, a flap not exceeding one-sixth of the circumference of the limb was elevated on each side of the incision to secure flap survival (23). Excessive thinning or dissection can cause skin damage. If an intramuscular lesion was considered resectable, radical excision was attempted. After dissection, exposure and separation of the lesion from the surrounding structures, radical or partial resection with adequate margins, was undertaken. Hemostasis and irrigation of the surgical field occurred before wound closure, and a suction drain was placed. Proper postoperative dressing and monitoring were performed. Important nerves, arteries, and veins were carefully separated and protected. If the contracture was complicated, a tendon length/transfer was performed. Neurolysis was performed when the vital nerves are involved (12). Additional technical details can be found in other reports (8, 19).

### Definition of operative safety, efficacy, and outcome

Intraoperative complications of major vessels and nerves, such as the femoral, popliteal, and posterior tibial vessels and sciatic, tibial, and common and deep peroneal nerves, were defined as complete or partial transection, perforation, split, or other gross injuries requiring surgical repair. Symptomatic CO_2_ gas or fat pulmonary embolism were considered major complications. Operative duration was defined as the time from incision initiation to the completion of postoperative dressing. Postoperative persistent bleeding via a drainage tube from the surgical field for > 5 days (>50 mL/day) requiring surgical hemostasis or transfusion of blood products was also considered major complication. Postoperative persistent lymph drainage was defined as lymph drainage >50 mL/day for > 5 days.

The lesion specimens were fixed in formalin and embedded in paraffin. All specimens were analyzed by two experienced pathologists at each center and evaluated microscopically for histopathological features, including nature (benign vs. malignant), lesion size, proliferative features, components, and depth. If a radical resection was performed, the resection margin status was classified as positive, negative, or indeterminate.

Other surgery-related complications, including intraoperative local skin burns, bidirectional conversion, postoperative bleeding, persistent lymph drainage, surgical site infection, wound dehiscence, flap necrosis, hematoma/seroma, persistent symptoms of superficial peroneal nerve injury, and local sensory paralysis, were also assessed. Wound dehiscence and flap necrosis requiring flap transfer to reconstruction were defined as major complications. Functional outcome evaluations included pain relief, contracture cure, and joint motion improvement. Normal joint motion referred to achieving the full, physiological range of motion for the joint. Near-normal joint motion was defined as ambulation (walking) that appeared functionally normal without limitation, despite the joint not achieving its full anatomical range of motion. For instance, a normal ankle joint can achieve dorsiflexion beyond 90 degrees (e.g., to approximately 20-25 degrees). An ankle with near-normal motion might achieve dorsiflexion to 90 degrees (neutral position), but cannot dorsiflex beyond this point (i.e., it lacks the normal additional range of ∼20-25 degrees).

## Statistical analysis

Statistical sample size calculations were not performed. Propensity score matching was used to minimize the differences in clinical characteristics between the SOFTES and OS groups. A logistic regression model was used to calculate the propensity scores based on age, lesion location, lesion size, and surgical site. One-to-one matching was employed between the SOFTES and OS groups using the nearest-neighbor method with a caliper of 0.2 width of the standard deviation of the propensity score. All statistical analyses were performed using the SciPy library in Python. Continuous variables were compared using the Mann–Whitney U test and summarized as medians with interquartile ranges or mean ± SD. Categorical variables were analyzed using Pearson's chi-square or Fisher's exact test. All tests were two-tailed, and a *p* value of <0.05 was considered statistically significant.

## Results

### Comparison of baseline characteristics before propensity score matching

We initially reviewed 414 patients who underwent soft-tissue lesion resection. In total, 150 patients (SOFTES, *n* = 75; OS, *n* = 75) were included in the final cohort (Figure 1 and Table 2). Surgical sites included the shin and calf (*n* = 111), thigh (*n* = 14), forearm (*n* = 9), gluteal region (*n* = 5), opisthenar (*n* = 2), chest wall (*n* = 2), face (*n* = 4), upper arm (*n* = 2), and back (*n* = 1) (Table 2).

**Table 2:** Baseline characteristics of patients before and after propensity score matching.

Characteristics	Before matching			After matching		
	SOFTES group (*n* = 125)	OS group (*n* = 194)	*p*-value	SOFTES group (*n* = 75)	OS group (*n* = 75)	*p*-value
Age at referal (years)	7 (1–38)	7 (0.5–64)	0.476	6 (1–38)	6 (1–38)	0.936
Lesion characteristics			0.039*			0.703
Subcutaneous layer/Deep fascia	62 (49.18%)	116 (56.21%)		44 (59.15%)	47 (54.17%)	
Intramuscular	63 (50.82%)	78 (43.79%)		31 (40.84%)	28 (35.42%)	
Tendon or Contracture	27 (21.31%)	66 (21.30%)		19 (21.13%)	24 (10.42%)	
Size of lesion (cm)**	15 (2-35)	15 (1-55)	0.0002*	15 (2-25)	15 (1-30)	0.728
Surgical sites			0.005*			0.830
Shin and calf	81 (64.80%)	88 (45.36%)		57 (76.00%)	55 (73.33%)	
Thigh	14 (11.20%)	22 (11.34%)		5 (6.67%)	9 (12.00%)	
Forearm	9 (7.20%)	14 (7.22%)		4 (5.33%)	5 (6.67%)	
Gluteal region	3 (2.40%)	6 (3.09%)		2 (2.67%)	2 (2.67%)	
Opisthenar	1 (0.80%)	6 (3.09%)		1 (1.33%)	1 (1.33%)	
Chest wall	2 (1.60%)	5 (2.58%)		1 (1.33%)	1 (1.33%)	
Face	3 (2.40%)	5 (2.58%)		2 (2.67%)	2 (2.67%)	
Upper arm	8 (6.40%)	3 (1.55%)		2 (2.67%)	0 (0.00%)	
Faciocervical region	0 (0.00%)	1 (0.52%)		0 (0.00%)	0 (0.00%)	
Neck	0 (0.00%)	8 (4.12%)		0 (0.00%)	0 (0.00%)	
Back	4 (3.20%)	12 (6.19%)		1 (1.33%)	0 (0.00%)	
Foot	0 (0.00%)	4 (2.06%)		0 (0.00%)	0 (0.00%)	
Tongue	0 (0.00%)	5 (2.58%)		0 (0.00%)	0 (0.00%)	
Lip	0 (0.00%)	1 (0.52%)		0 (0.00%)	0 (0.00%)	
Palm	0 (0.00%)	4 (2.06%)		0 (0.00%)	0 (0.00%)	
Perineum	0 (0.00%)	2 (1.03%)		0 (0.00%)	0 (0.00%)	
Ear	0 (0.00%)	2 (1.03%)		0 (0.00%)	0 (0.00%)	
Sub-scalp	0 (0.00%)	6 (3.09%)		0 (0.00%)	0 (0.00%)	

*n* (%), median (Range); SOFTES, soft tissue endoscopic surgery; OS, open surgery.

*p < 0.05; **In patients with extensive lesions, such as extensive microcystic lymphatic malformation, the size of lesion referred to the maximum diameter of the plan-to-resect lesion per operation.

The baseline characteristics of the eligible patients before and after matching are shown in Table 2. Patients in the SOFTES group had significantly smaller lesion size [15 cm (2-35) vs. 15 cm (1-35); *p* < 0.005] than those in the OS group before matching. There were statistically significant differences in the sites of surgery (*p* = 0.005) and lesion depth (subcutaneous layer/deep fascia, intramuscular, tendon, or contracture; *p* = 0.039) between the groups.

### Comparison of operative data after propensity score matching

Seventy-five pairs were matched. The baseline characteristics were well balanced between the two groups after matching (Table 2). The treatment outcomes of the groups after matching are shown in Table 3. Compared with the OS group, the estimated blood loss in the SOFTES group was significantly lower [20 mL (1-500) vs. 50 mL (5-600); *p* = 0.001]; however, the operative duration was significantly longer [223 minutes (35-490) vs. 173 minutes (37-494); *p* = 0.008].

**Table 3:** Matching factors and treatment outcomes between the sOFTES and OS groups after propensity score matching

Outcomes	SOFTES group (*n* = 75)	OS group (*n* = 75)	*p*-value
Major vessels complications	0 (0)	0 (0)	NA
Major nerves injury	0 (0)	0 (0)	NA
Symptomatic gas or fat pulmonary embolism	0 (0)	0 (0)	NA
Facial nerve injury	0 (0)	0 (0)	NA
Estimated blood loss (mL)	20 (1-500)	50 (1-600)	0.001*
Operative duration (minutes)	223 (35-490)	173 (37-494)	0.008*
Local burn of skin (<2 cm^2^)	4 (5.33%)	0 (0.00%)	0.048*
Superficial peroneal nerve injury	2 (2.67%)	1 (1.33%)	0.568
Bi-directional approach**	4 (5.33%)	4 (5.33%)	1.000
Postoperative bleeding	1 (1.33%)	3 (4.00%)	0.324
Persistent lymph drainage	2 (2.67%)	3 (4.00%)	0.660
Local sensory paralyses (<5 cm^2^)	5 (6.67%)	3 (4.00%)	0.491
Surgical site infection	1 (1.33%)	2 (2.67%)	0.568
Wound dehiscence	0 (0.00%)	7 (9.33%)	0.010*
Flap necrosis	1 (1.33%)	11 (14.67%)	0.005*
Hematoma/Seroma	4 (5.33%)	4 (5.33%)	1.000
Residual mild pain	3/33	7/40	0.363
Cure of contracture	19/19	23/24	0.922
Normal or near a normal joint motion	33/33	38/38	NA

*n* (%) or mean ± standard deviation or median (range); SOFTES, soft tissue endoscopic surgery; OS, open surgery.

*p < 0.05; **Bi-directional approach refers to endoscopy-assisted small-incisional surgery, technical details in (13).

### Comparison of intra- and postoperative complications

No major vessel or nerve complications, or severe gas/fat pulmonary embolisms occurred in either group (Table 3). The incidence of local burn of the skin was slightly higher in the SOFTES group compared to the OS group (*p* = 0.048). The incidences of superficial peroneal nerve injury, postoperative bleeding, persistent lymph drainage, local sensory paralyses (<5 cm^2^), surgical site infection, hematoma/seroma, and residual mild pain were not significantly different between the two groups. However, the incidence of wound dehiscence [*n* = 0 (0.00%) vs. *n* = 7 (9.33%); *p* = 0.010] and flap necrosis [*n* = 1 (1.33%) vs. *n* = 11 (14.67%); *p* = 0.005] was significantly higher in the OS group than in the SOFTES group.

### Managements, outcomes, and follow-up of complications

Some postoperative bleeding required transfusion of blood products (Table 4). Wound dehiscence, flap necrosis, and focal burns of the skin were managed with a change in fresh dressing for the wound, re-suturing, or flap transfer; however, scarring was usually obvious (Figure 3). Peripheral nerve rehabilitation was performed for sensory nerve injury. Persistent lymph drainage was managed using sclerotherapy via a draining tube with ethanol and/or bleomycin. Surgical site infection was treated with systemic and/or topical antibiotics and a fresh dressing. Two patients with subcutaneous hematoma/seroma were treated with puncture and aspiration with elastic compression. In other patients, subcutaneous bleeding or effusion from the surgical field spontaneously resolved approximately 1-2 weeks after surgery. Rehabilitation training started 2-3 weeks after surgery in patients with limited joint motion. Typically, residual pain is difficult to treat. If residual lesions were unresectable, such as intranervous VM or FAVA infiltrating major nerves, genetic testing and targeted therapy was considered using the mTOR inhibitor sirolimus or *PI3 K* inhibitor alpelisib. Recontraction is often caused by residual pain due to the FAVA, venous thrombosis, and fibrosis. Endoscopic resection or recession was performed to avoid recontraction owing to extensive adhesions. There was obvious scarring and suture marks in the OS group, especially in patients with wound dehiscence, flap necrosis, and focal skin burns. Wound dehiscence was not observed in the SOFTES group.

**Table 4:** Managements and outcomes of complications after both surgical approaches.

Complications	Managements		Outcomes
	SOFTES group	OS group	
Postoperative bleeding	One patient required red cell transfusion	One patient required red cell transfusion; 3 patients required albumin transfusion	Cure
Wound dehiscence	0	Change fresh dressing for the wound; Re-suture	Cure with scarring
Local burn of skin (<2 cm^2^)	Change fresh dressing for the wound; Suture		Cure with scarring
Superficial peroneal nerve injury	Peripheral nerve rehabilitation		Improved or cure
Persistent lymph drainage	Sclerotherapy via the draining tube with ethanol and/or bleomycin		Cure
Local sensory paralyses (<5 cm^2^)	Peripheral nerve rehabilitation for months		Improved or recovery of sense
Surgical site infection	Systemic and/or topical antibiotics; Change fresh dressing for the wound		Cure
Flap necrosis	Change fresh dressing for the wound; Flap transfer		Cure with scarring
Hematoma/Seroma	Two patients underwent puncture and aspiration with elastic compression.		Cure
Residual pain	If residual lesion was resectable, re-resection was done. If it was unresectable, such as venous malformation or FAVA infiltrating major nerves, genetic test and targeted therapy could be considered, using mTOR inhibitor sirolimus or *PI3 K* inhibitor alpelisib.		Significant relief or cure
Re-contracture	Endoscopic resection of residual lesion or recession to avoid extensive adhesion		Normal or near-normal motion

SOFTES, soft tissue endoscopic surgery; OS, open surgery.

All patients were followed up for 11 months to 5 years (median, 23 months). No recurrence was observed in either group for patients with benign tumors (*n* = 8). However, residual lesions persisted in patients with extensive vascular malformations and primary lymphedema. This outcome was expected, as the surgical aim in these patients was debulking rather than radical resection. Consequently, planned staged surgeries were required for these patients. Residual pain relief or significant improvement was reported by patient/parent, as did contractures in all patients. All patients had normal or near-normal joint motion range (Table 4).

## Discussion

The use of endoscopic surgery has evolved from luminal cavities to SOFTES in recent years (8–13, 19, 21, 22, 24–27). The evolution of endoscopic approaches has progressed through distinct phases. First, endoscopy was used for traditional interventions within natural body lumens [gastrointestinal tract (28), airways (29), genitourinary system (30, 31)]. Second, over the last two decades, endoscopy has been applied for surgeries in peritoneal (32), thoracic (33), ventricular (34), cardiac (35), and articular spaces (36, 37). For example, natural orifice transluminal endoscopic surgery is an incision-free procedure, in which the peritoneal cavity is endoscoped through natural orifices via a planned incision in the stomach, vagina, or colon (26, 38–40). Third, endoscopy using advanced techniques (41), has been used for intramural/submucosal interventions, and fourth, soft tissue endoscopy has emerged more recently. The term soft tissue endoscopy was proposed in 2016 (21). We proposed the concept of a fourth surgical space (13) in which the workspace is created or developed in the subcutaneous layer and inter/intramuscular space to perform SOFTES (8, 12, 13). This potential field between the integumentary and musculoskeletal systems offers substantial unexplored possibilities for SOFTES beyond the current open approach (21).

The versatility of SOFTES is demonstrated through use for classic transaxillary thyroidectomy (42, 43) and endoscopic breast surgery (44); applications in benign lesion excision [dermoids, osteomas, lipomas (10, 20, 45, 46)], tenoscopy for synovial or ganglion cysts and ganglionectomy (47–49); advanced musculoskeletal surgeries [such as transverse Vulpius gastrocsoleus recession, intramuscular and aponeurotic Achilles tendon lengthening (50), and FAVA resection (1, 8, 12, 13)]; and various vascular tumor and malformations, and related complications management (1, 8, 12, 13).

Compared to conventional OS, recent comparative studies and our propensity score matched comparison (13, 19) confirmed significant SOFTES advantages, including minimal incision size (“stealth” incisions) (10, 20), magnified visual field under endoscopy, less blood loss, accelerated recovery time, shorter hospital stay, reduction in wound healing time, higher rate of patient satisfaction, equivalent complication rates despite technical complexity, and better aesthetic outcomes from concealed scars. SOFTES can also provide symptom relief, debulking, functional reconstruction/improvement, and less postoperative pain (12, 13). However, the SOFTES group had a longer operative duration and a slightly higher incidence of focal skin burns than the OS group. An exceptionally long operative duration is usually attributable to the large size, deep location of the lesion, and prolonged retrieval times (10, 12, 20). Burns were induced by a light source, monopolar cautery, or an ultrasonic scalpel.

Lesion characteristics that correlate with safety and effectiveness include depth-to-size ratio, anatomical accessibility, tissue plane integrity, and FAVA staging (8, 13). Posterior tibialis and vastus intermedius lesions present visualization challenges and often require endoscopy-assisted mini-open approaches (13). Tourniquet use is also an important factor that can prevent intraoperative bleeding and fat or gas pulmonary embolism (12, 13).

Both OS and SOFTES require a learning curve, particularly SOFTES. The learning curve components for SOFTES include endoscopic fundamentals, spatial orientation in soft tissue planes, maintenance of endoscopic hemostasis, endoscopic vessel/nerve separation, and specimen retrieval optimization. Initial cases required prolonged retrieval times through sub-centimeter incisions (10, 12, 20). Strategic incision enlargement (15–20 mm) reduces the retrieval duration without compromising aesthetic outcomes (13). It is necessary to develop endoscope-based operating instruments to aid in this step of the surgery (10, 20).

Preliminary comparisons between SOFTES and OS before matching were not performed because of fundamental differences in the case selection criteria. Our center typically reserves OS for complex presentations (larger lesion size, deeper anatomical involvement, vital structure infiltration, or multifocal disease), whereas SOFTES is predominantly applied to discrete superficial or smaller pathologies. This inherent selection bias precluded a direct comparison of the operative duration and complication rates between the approaches. We believe that the operative duration will be reduced with more experience.

Future technical innovations should focus on the development of dedicated SOFTES instrumentation, enhanced visualization systems for deep muscular planes, retrieval systems with integrated morcellation, flexible specimen containment bags for this approach to soft tissue malignancies, and expansion into oncological applications with marginal control. Hybrid approaches combining endoscopic and limited-open techniques (bidirectional approach or endoscopy-assisted open surgery) (13) and single-site endoscopic surgery should also be developed (1, 9) because more difficult surgeries can be completed through both approaches, such as FAVA involving the Hunter's canal (1).

### Limitations

The study had some limitations because of its retrospective nature and the heterogeneity of conditions and surgeries. Because the indications for SOFTES and OS are different, and lesions vary significantly in their manifestation and extent, a sufficient prospective comparison between SOFTES and OS would be difficult to accomplish at our center. We applied propensity score matching, the gold standard methodology in observational research for addressing confounding biases when evaluating therapeutic interventions, to form balanced cohorts through a counterfactual framework analysis. This method simulates a prospective randomized controlled trial to minimize selection bias as much as possible. However, it is not a prospective randomized controlled trial. As a retrospective study, selection bias cannot be completely eliminated; we can only strive to minimize it. OS is more suitable for more complex diseases; therefore, SOFTES cannot entirely replace traditional OS. Normal or near-normal joint motion was obtained, which may be due to patients with less complex lesions after matching. The follow-up duration was limited because of the complexity of vascular malformations and symptom control, and disease recurrence.

## Conclusions

SOFTES is a valuable addition to the soft tissue surgeons’ armamentarium, particularly when prioritizing aesthetic outcomes and accelerated recovery. Future randomized controlled trials using matched cohorts are required to better delineate comparative safety and effectiveness. Our study broadens the spectrum of applications of endoscopic surgery for various soft tissue diseases. Technological refinement will expand the therapeutic scope while maintaining the fundamental advantages of endoscopic surgery, including oncological, general, pediatric, vascular, plastic, dermatological, and orthopedic surgeries.

## Data Availability

The raw data supporting the conclusions of this article will be made available by the authors, without undue reservation.
